# Expression of *Rickettsia* Adr2 protein in *E*. *coli* is sufficient to promote resistance to complement-mediated killing, but not adherence to mammalian cells

**DOI:** 10.1371/journal.pone.0179544

**Published:** 2017-06-29

**Authors:** Daniel A. Garza, Sean P. Riley, Juan J. Martinez

**Affiliations:** Vector-Borne Diseases Laboratories, Department of Pathobiological Sciences, Louisiana State University School of Veterinary Medicine, Baton Rouge, Louisiana, United States of America; University of Helsinki, FINLAND

## Abstract

Bacteria exposed to host serum are subject to the antibacterial effects to the complement system. However, pathogenic microorganisms have evolved mechanisms of evading this immune attack. We have previously demonstrated that at least two *R*. *conorii* antigens, RC1281/Adr1 and OmpB β-peptide, contribute to the evasion of complement-mediated killing by binding the complement regulatory proteins vitronectin and factor H. RC1282/Adr2, a protein related to Adr1, is predicted to share similar structural features, suggesting that this protein may also contribute to evasion of complement-mediated killing. Interestingly, the *R*. *prowazekii* Adr1 and Adr2(RP828) proteins were originally found to interact with host cell surface proteins, suggesting their putative roles as adhesins in this pathogenic rickettsial species. In this study, we expressed both *R*. *conorii* and *R*. *prowazekii* Adr2 on the surface of a non-adherent, serum-sensitive strain of *E*. *coli* to examine the potential role of this protein to mediate evasion of complement-mediated killing and adherence to host cells. We demonstrate that, similar to *R*. *conorii* Adr1, *R*. *conorii* and *R*. *prowazekii* Adr2 are sufficient to mediate serum resistance and to promote interaction with the host complement regulator vitronectin. Furthermore, we demonstrate that expression of Adr2 in a non-adherent strain of *E*. *coli* is insufficient to mediate adherence to cultured mammalian endothelial cells. Together, our data demonstrate that the *R*. *conorii* and *R*. *prowazekii* Adr2 protein does not participate in the interactions with mammalian cells, but rather, participates in the evasion of killing by complement.

## Introduction

*Rickettsia conorii* is a small Gram-negative, obligate-intracellular bacterium, and the etiological agent of endemic Mediterranean spotted fever (MSF). MSF is transmitted by an arthropod vector, and subsequent dissemination of the microbe throughout a human host results in the development of a characteristic maculopapular dermal rash [1]. Although this disease is commonly referred to as a milder version of the spotted fever group rickettsioses, lack of timely medical treatment can prove fatal [2]. Seroprevelance in affected canine populations despite increased use of anti-tick treatments, cyclic re-emergence of disease, and risk of fatal clinical complications in infected persons demonstrate that this pathogen is an active threat to human health [3–5].

Rickettsial pathogens must employ several mechanisms to persist in the presence of opsonophagocytosis or host bactericidal complement-mediated lysis while in blood circulation, during invasion of target cells, and subsequent intracellular proliferation. Spotted fever group (SFG) *Rickettsia* sp. express a multitude of membrane-bound proteins which have been demonstrated not only to play integral roles in rickettsial pathogenesis through processes of adherence and invasion of host cells, but confer resistance to anti-bacterial host responses [6–14]. Among the characterized outer membrane proteins, *Rickettsia conorii* RC1281/Adr1 was demonstrated to recruit host regulatory complement protein vitronectin in order to confer resistance to serum-mediated killing [9]. Its paralog, Adr2(RC1282), has been identified in all pathogenic species in the genus *Rickettsia*, and is implicated in the entry of *R*. *prowazekii* into non-phagocytic mammalian cells [12, 15]. However, its putative role in SFG rickettsiae, such as *R*. *conorii*, has yet to be elucidated.

We have previously demonstrated that *R*. *conorii* remains viable in the presence of serum complement proteins, and that depletion of one of these regulatory proteins, such as factor H and vitronectin, is not sufficient to completely ablate serum sensitivity [10, 11]. These findings indicate that mechanisms of redundancy exist within the rickettsial genome, and additional membrane bound proteins may contribute in the serum-resistance phenotype in a similar fashion.

## Results

### Adr2 is conserved among pathogenic species of Rickettsia

**Fig 1:**
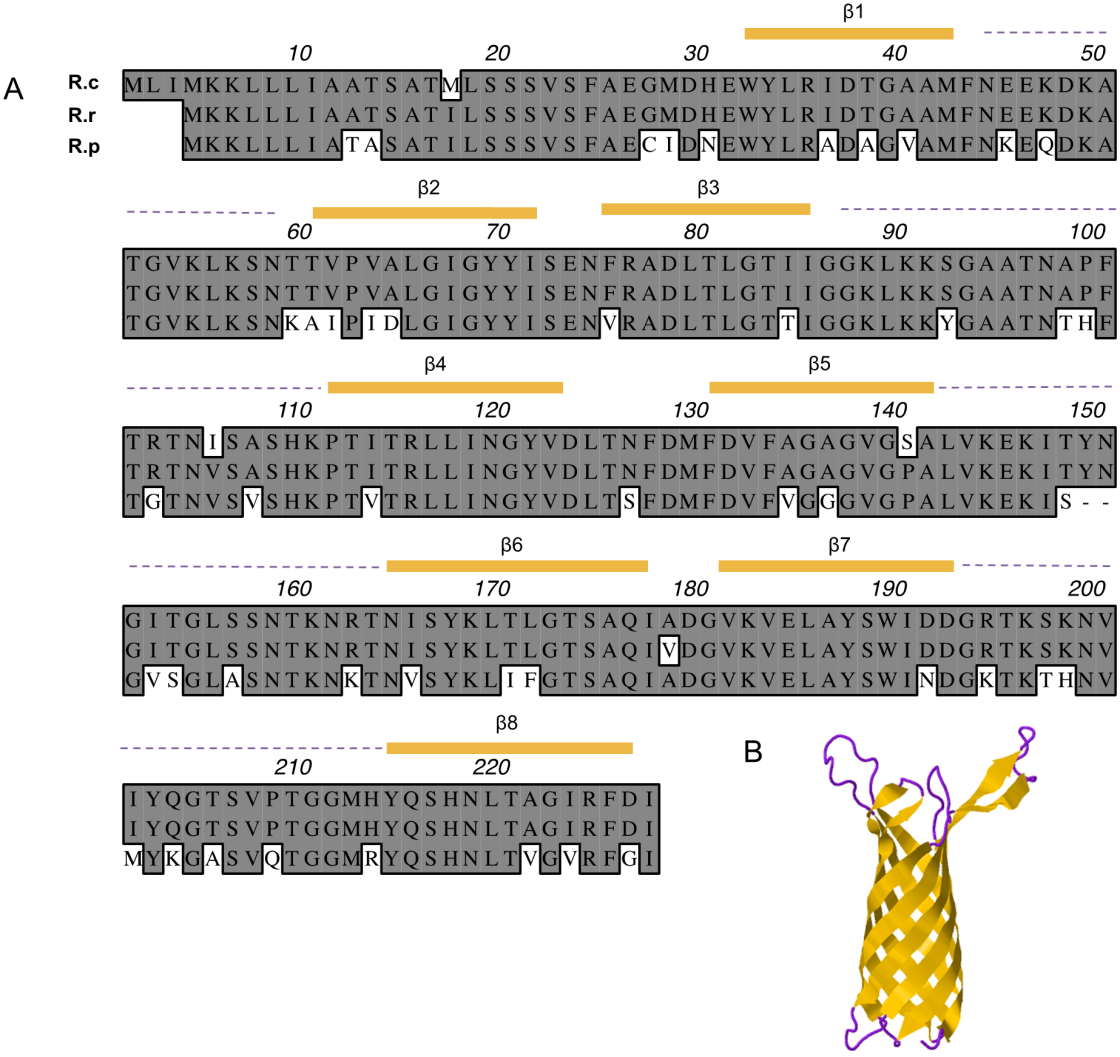
Adr2 is highly conserved among pathogenic rickettsial species. (A) ClustalW alignment of Adr2 homologs from rickettsial species in both Spotted Fever Group (*R*. *conorii*, *R*. *rickettsii*) and Typhus Group (*R*. *prowazekii*). Predicted β-sheets and surface exposed loops are indicated in yellow and purple dashed lines, respectively. (B) Phyre2 predicted structure of *R*. *conorii* Adr2 demonstrates of 8 transmembrane β-sheets configured in a “barrel”-like structure with possible surface exposed loops.

Alignment of amino acid profiles for *R*. *conorii*, *R*. *rickettsii*, and *R*. *prowazekii* Adr2 is shown in Fig 1A. Analysis of *R*. *conorii* Adr2 sequence demonstrated sequence similarities to proteins belonging to the superfamily OMPA [16]. These proteins are characterized by having trans-membrane beta-barrels, which are thought to form a pore-like structure at the outer membrane [17]. The Phyre2 protein structure-modeling algorithm predicts that two of the highest confidence structural models are based on threading against the *N*. *gonorrhea* NspA protein (PDB: d1p4ta) and the Opa60 protein (PDB: c2nlha). The model for *R*. *conorii* Adr2 predicts a protein that contains 8 outer membrane-spanning β-sheets and 4 surface exposed peptides (Fig 1B). Although the similarity between the deduced amino acid sequences of Adr1 and Adr2 in *R*. *conorii* is approximately 41%, both predicted structures based on the Phyre2 algorithm are virtually identical, suggesting that both proteins may share functional similarities [15].

### Adr2 is present at the *R*. *conorii* outer membrane

**Fig 2:**
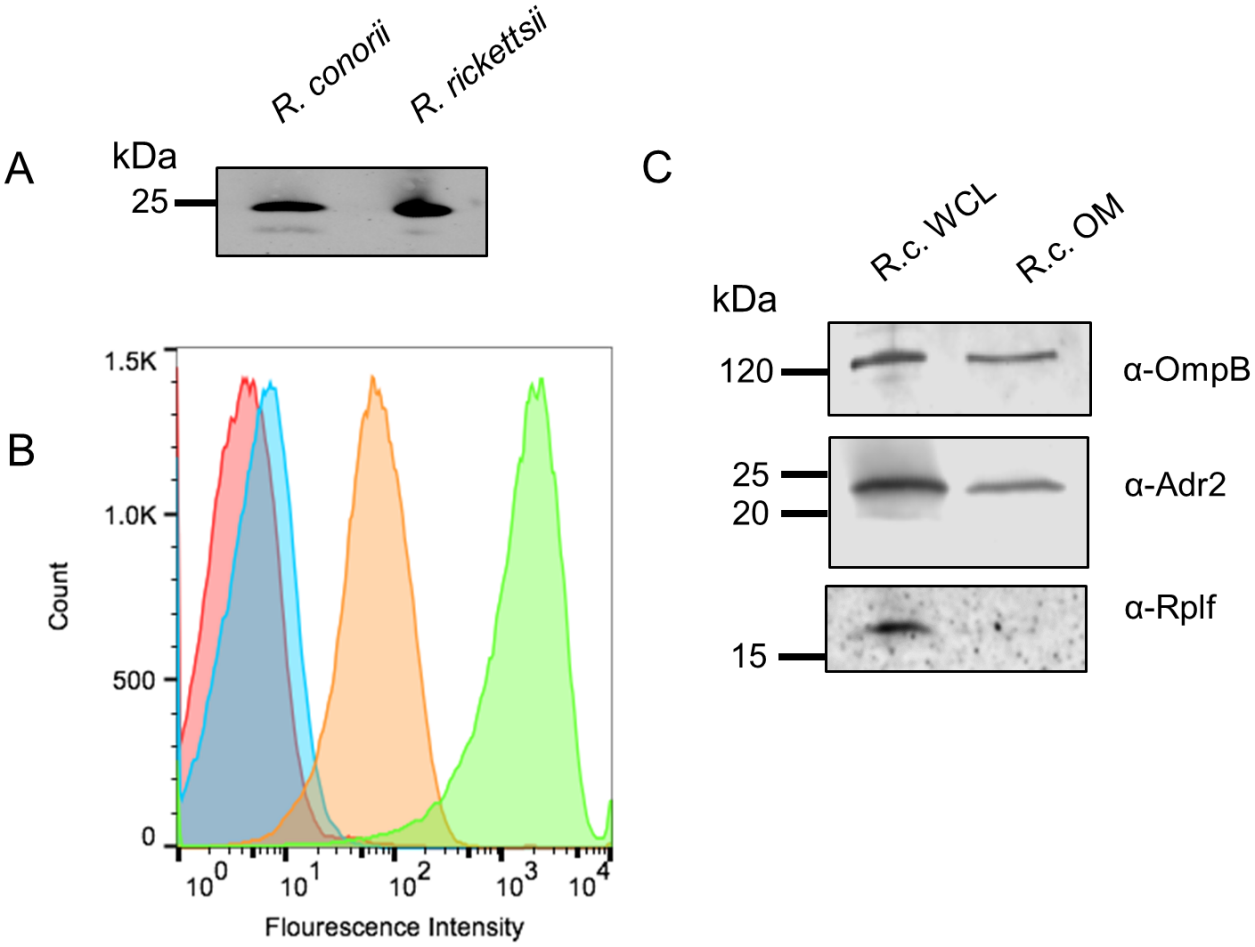
Outer-membrane expression of Adr2 in *R*. *conorii*. (A) Western blot analysis using rabbit-anti-Adr2 antiserum confirms the expression in *R*. *conorii* Malish 7 and *R*. *rickettsii* Sheila Smith whole cell lysate (WCL). (B) Flow cytometry confirmed the expression of Adr2 at the surface of *R*. *conorii*. A shift in fluorescence is observed when fixed *R*. *conorii* was incubated with primary and secondary antibodies (orange) compared to samples prepared with only primary or secondary (red and blue, respectively). A sample is incubated with Anti-*R*. *conorii* serum (green) as a positive control of the flow cytometer. (C) Western blot analysis of *R*. *conorii* whole cell lysates (WCL) and outer-membrane(OM) preparations using antibodies against OmpB (mAb 6B6.6) or Adr2 demonstrates the presence of reactive species in both WCL and OM preparations. Anti-RplF (50s ribosomal protein L6) is used a control for cytoplasmic contents.

To demonstrate that Adr2 is surface exposed in *R*. *conorii*, we produced polyclonal antiserum raised against the predicted surface exposed domains termed loops 1 and 2. Western immunoblot analysis of both *R*. *conorii* Malish 7 and *R*. *rickettsii* Sheila Smith whole cell protein lysates (WCL) yielded reactive bands at approximately 25 kDa for both species (Fig 2A). To verify surface expression of Adr2, paraformaldehyde-fixed *R*. *conorii* cells were stained with anti-Adr2 antibody and a fluorophore-tagged secondary antibody. As shown in Fig 2B, flow cytometric analysis of stained bacteria demonstrated a significant shift in fluorescence intensity (orange trace) compared to untreated bacteria (red trace) or *R*. *conorii* labeled with secondary antibody only (blue trace). Additionally, we verified expression of Adr2 in the rickettsial outer membrane by western-blot analysis of isolated *R*. *conorii* outer membrane fractionations (Fig 2C). Taken together, these results indicate that Adr2 is present at the surface of *R*. *conorii* and that segments of this protein are exposed to the extracellular environment.

### Adr2 mediates resistance to serum killing in complement

**Fig 3:**
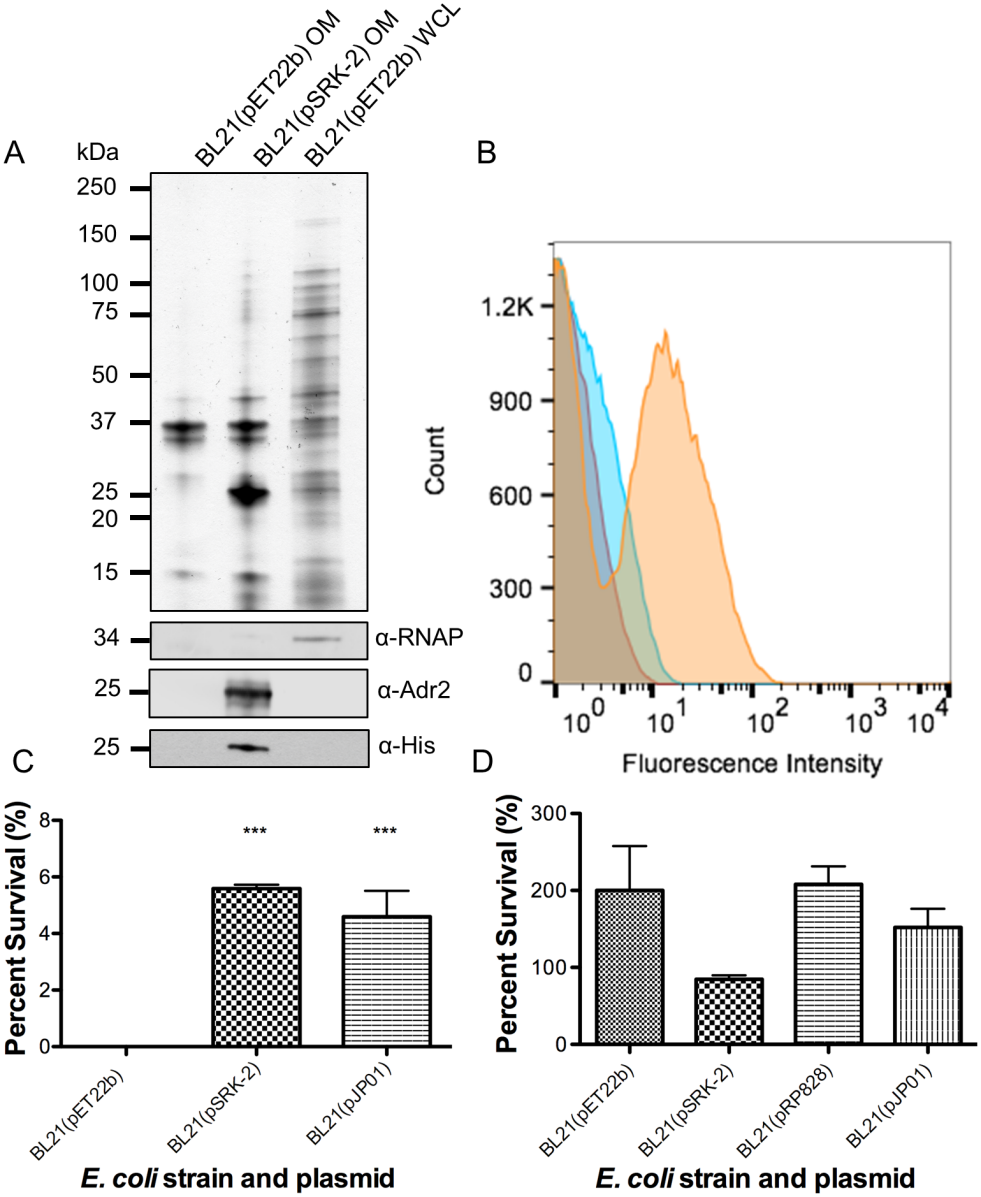
Expression of Adr2 at the *E*. *coli* outer-membrane is sufficient to mediate resistance to serum-killing. (A) OM preparations or WCL from *E*. *coli* transformed with the empty vector (pET22b) or the plasmid encoding for *R*. *conorii* His_6_-tagged Adr2 are separated by SDS-PAGE. Subsequent silver staining demonstrates similar protein loading and migration. Anti-*E*. *coli* RNA Polymerase alpha (RNAP) is used to demonstrate the presence of cytoplasmic contents. (B) Flow cytometry analysis reveals the expression of Adr2 at the surface of *E*. *coli*. A shift in fluorescence was observed in BL21(pSRK-2 [*R*. *conorii* Adr2]) in cultures incubated with both primary and secondary antibodies (orange) compared to primary(red) or secondary(blue) alone. (C) Expression of *R*. *conorii* Adr2(pSRK-2) in *E*. *coli* (BL21) is sufficient to mediate serum-killing to normal human serum. *R*. *conorii* Adr1(pJP01) is used as a positive control. Results are shown as the mean ± SD (P values: *** = 0.0007). (D) Serum-sensitive *E*. *coli* (pET22b) and cultures expressing Adr2 or Adr1 survive when incubated with heat-inactivated normal human serum.

To determine if *R*. *conorii* Adr2 is expressed at the outer-membrane of *E*. *coli*, we transformed a non-adherent, serum-sensitive strain of *E*. *coli* (BL21 (DE3)) with pSRK-2, a pET22b derivative containing an IPTG-inducible promoter driving expression of *R*. *conorii adr2* with a C-terminal His_6_ tag. Expression of Adr2 in transformed *E*. *coli* was verified by the presence of an immune reactive species at the predicted molecular weight within isolated outer membrane (OM) protein fractions (Fig 3A). In addition, surface expression of Adr2 in *E*. *coli* was verified by flow cytometry using anti-Adr2 antibodies directed against predicted extracellular domains (Fig 3B). These results demonstrate that expression of Adr2 in *E*. *coli* results in proper localization of this protein to the outer-membrane.

To demonstrate whether the expression of Adr2 could mediate serum resistance, we incubated cultures of Adr2-expressing *E*. *coli* with either PBS as a control or normal human serum (NHS) in PBS. Resistance to serum killing was measured by a colony forming unit (CFU)-based assay and is represented as the percentage of bacteria that survive serum challenge versus the same inoculum incubated in PBS [10]. As seen in Fig 3C, bacteria expressing Adr2 were significantly resistant to serum killing compared to bacteria harboring the empty vector. The observed survival was comparable to those previously observed for *R*. *conorii* Adr1-expressing *E*. *coli* (Fig 3C) and Riley *et al*., 2014. In contrast, bacteria harboring the empty vector as well as those expressing Adr2 and Adr1 survived when incubated with heat-inactivated NHS (Fig 3D). These results demonstrate that similar to Adr1, Adr2 is sufficient to confer resistance to serum killing when expressed at the outer membrane of *E*. *coli*.

### Adr2-expressing *E*. *coli* binds host complement regulatory protein vitronectin

**Fig 4:**
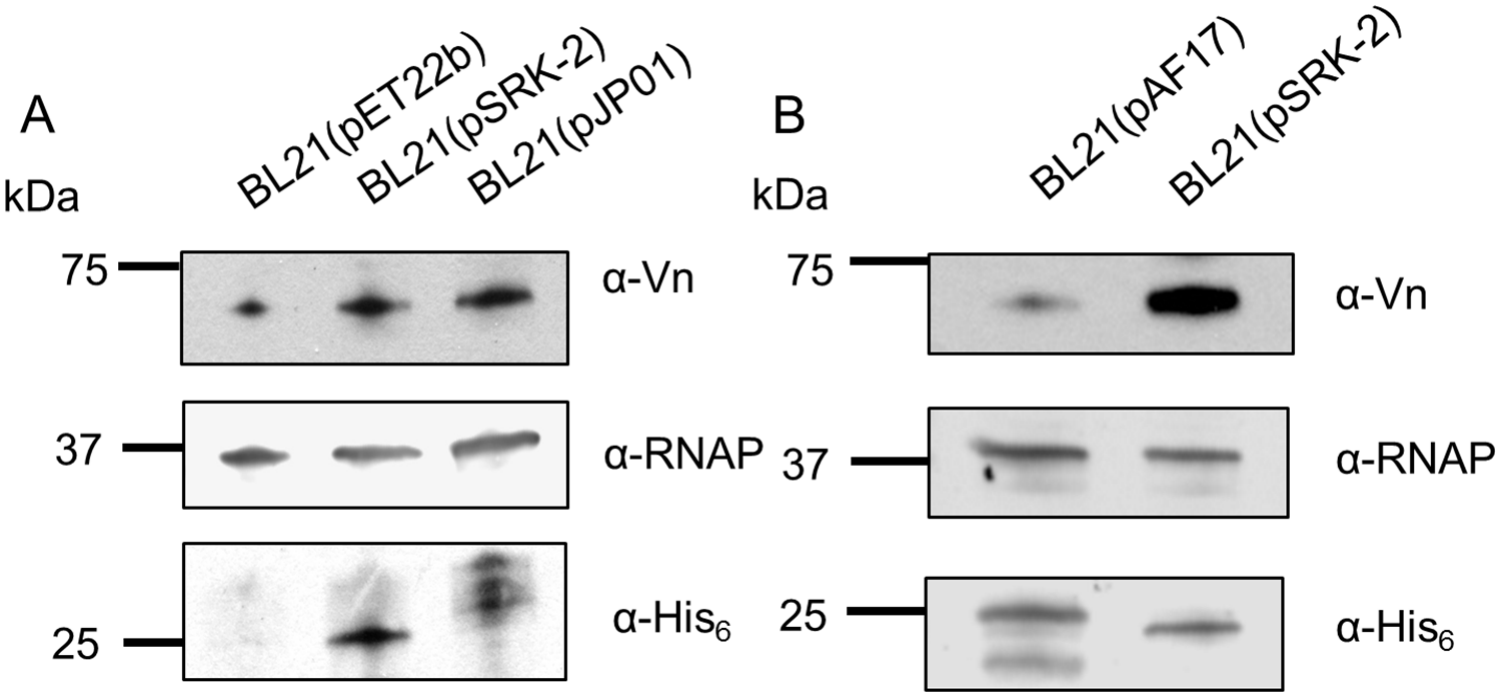
*R*. *conorii* Adr2 binds complement regulatory protein vitronectin. (A) *E*. *coli* BL21 harboring the empty vector pET22b, the plasmid encoding *R*. *conorii* His_6_-tagged Adr2 (pSRK-2) or *R*. *conorii* His-tagged Adr1 (pJPO1) were incubated with normal human serum. Western immunoblot analysis revealed a reactive band at approximately 75 kDa in all lanes except for the empty vector when probed with antibody raised against vitronectin. Expression of both Adr2 and Adr1 was confirmed by anti-His_6_, and equal loading was demonstrated with anti-*E*. *coli* RNA Polymerase alpha (RNAP). (B) Western immunoblot analysis of multimeric vitronectin binding to *E*. *coli* expressing Adr1 derivative mutant (pAF17) or *R*. *conorii* Adr2. Expression of both the Adr1 derivative and Adr2 was confirmed by anti-His_6_, and equal loading was verified using anti-*E*. *coli* RNA polymerase alpha (RNAP).

We next sought to determine how Adr2 contributes to the observed serum-resistance phenotype. We hypothesized that based on structural and functional similarities to Adr1, Adr2 would similarly bind a soluble complement regulatory protein such as vitronectin. To test this hypothesis, we incubated Adr2-expressing *E*. *coli* with NHS, co-sedimented bacteria and any interacting proteins, washed away any unbound proteins with PBS, and then prepared whole cell protein lysates. Bacterial proteins and any interacting serum proteins were separated by SDS-PAGE. Western immunoblot analysis revealed a reactive species at approximately 75 kDa in lysates of Adr2-expressing *E*. *coli*, but not in control *E*. *coli* lysates harboring the empty vector (Fig 4) when probed with an anti-vitronectin antibody. These results demonstrate that Adr2 interacts with vitronectin and this acquisition correlates to the observed serum-resistance phenotype.

**Fig 5:**
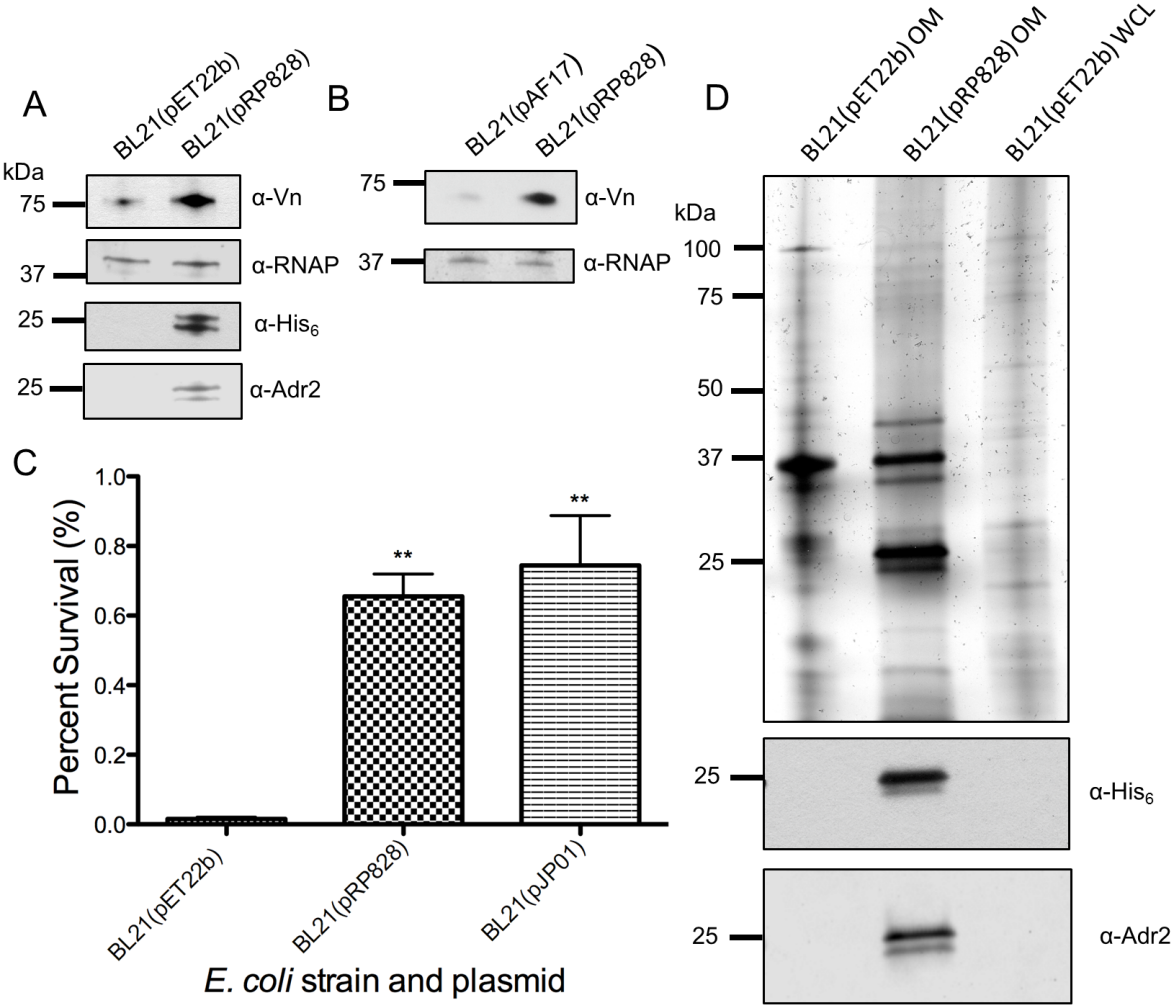
*R*. *prowazekii* Madrid E Adr2 confers acquisition of vitronectin and resistance in serum. (A) *E*. *coli* BL21(DE3) harboring the empty vector pET22b or the plasmid encoding *R*. *prowazekii* Madrid E His_6_-tagged Adr2 (pRP828) were incubated with normal human serum. Western immunoblot analysis revealed a reactive band at approximately 75 kDa in the lane with *R*. *prowazekii* Adr2 only when probed with antibody raised against vitronectin. Expression of Adr2 was confirmed by anti-His_6_, and equal loading was demonstrated with anti-*E*. *coli* RNA Polymerase alpha (RNAP). (B) Western immunoblot analysis of multimeric vitronectin binding to *E*. *coli* expressing Adr1 derivative mutant (pAF17) or *R*. *conorii* Adr2. Expression of both the Adr1 derivative and Adr2 was confirmed by anti-His_6_, and equal loading was verified using anti-*E*. *coli* RNA polymerase alpha (RNAP). (C) Expression of *R*. *prowazekii* Adr2 in *E*. *coli* BL21(DE3) is sufficient to mediate resistance in the presence of serum. Results are shown as the mean ± SD (P value: ** = 0.0024) and *E*. *coli* harboring *R*. *conorii* Adr1 (pJP01) was used as a positive control. (D) OM preparations or WCL from *E*. *coli* transformed with the empty vector (pET22b) or the plasmid encoding for *R*. *prowazekii* His_6_-tagged Adr2 are separated by SDS-PAGE. Subsequent silver staining demonstrates similar protein loading and migration. Anti-His_6_ was used to demonstrate expression of *R*. *prowazekii* Adr2. A reactive band at approximately 25 kDa was observed when probed with anti-*R*. *conorii* Adr2.

As seen in the amino acid alignment of *R*. *conorii* Adr2 and its homologs, sequence homology is greater amongst pathogenic SFG rickettsial species, but is still significant amongst members of the TG rickettsiae, including *R*. *prowazekii* (97.8% to 88.1% respectively). We hypothesized that because of this putative protein structural conservation, the *R*. *prowazekii* Adr2 protein would be sufficient to interact with Vn and provide resistance to complement-mediated killing. As predicted, Vn acquisition and serum resistance was observed at levels comparable to *R*. *conorii* Adr2 (Fig 5A and 5C). We further assessed the acquisition of Vn by determining whether Adr2 could directly bind Vn in absence of serum proteins. Cultures expressing Adr2, and *E*. *coli* harboring pAF17, a plasmid encoding for a non-Vn-binding Adr1 derivative, were incubated with multimeric Vn and whole cell lysates were subsequently prepared, separated by SDS-PAGE, and analyzed by western blot [18]. Probing with an anti-vitronectin antibody revealed a reactive band at approximately 75 kDa in lysates of Adr2 expressing *E*. *coli*, but not in the lanes containing pAF17 lysate (Figs 4B and 5B). These results demonstrate that direct Vn binding by Adr2 is a phenotype conserved across both SFG and TG rickettsial species.

### Recombinant *E*. *coli* expressing *R*. *conorii* Adr2 are not adherent to mammalian cells

**Fig 6:**
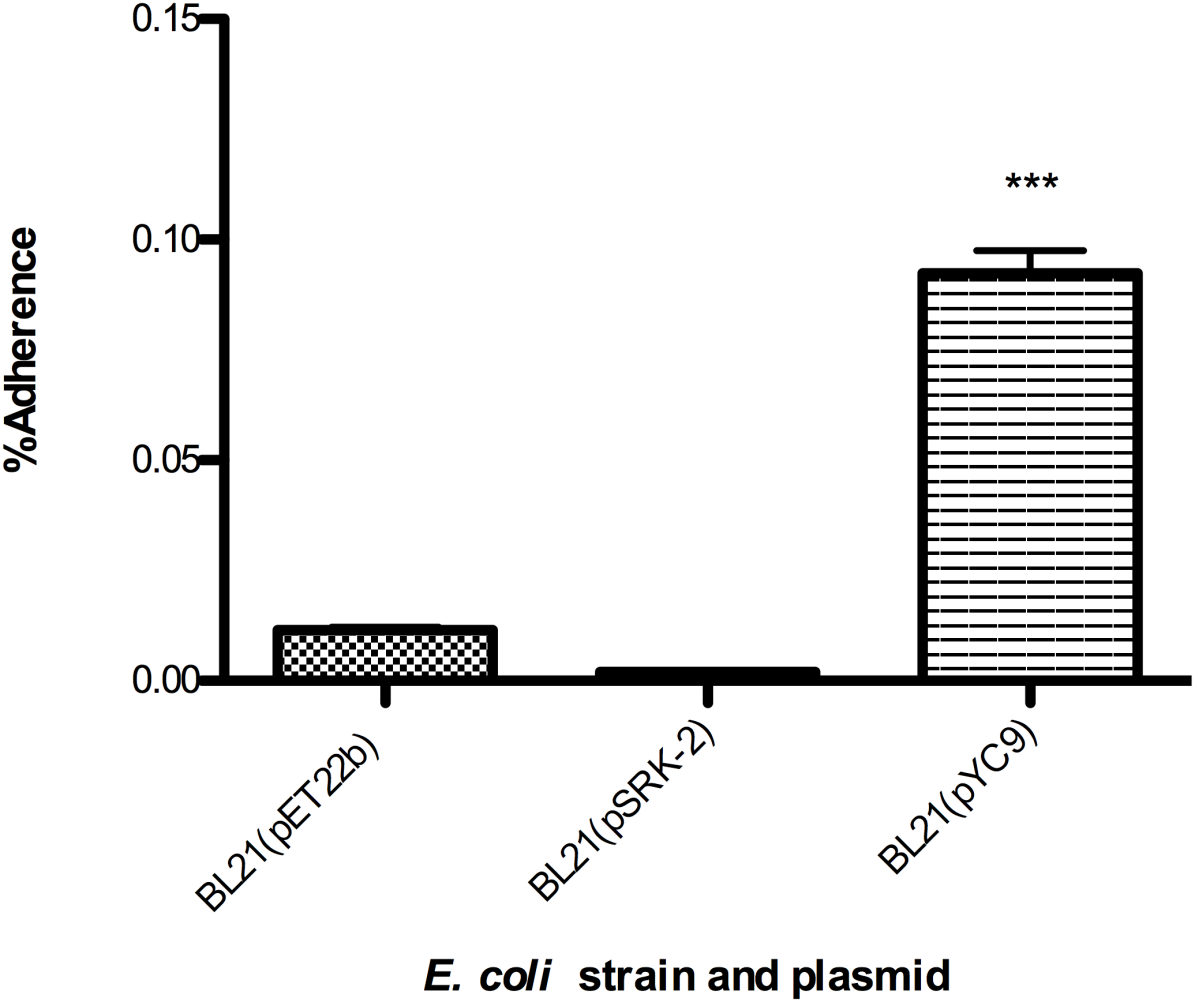
Adherence potential of *R*. *conorii* Adr2 expressing *E*. *coli*. Expression of *R*. *conorii* Adr2 in *E*. *coli* (BL21) is not sufficient to mediate cell adherence to EA.hy926 cells. The results are expressed as percentages of recovered adherent bacteria based on the total bacterial input. Results are shown as the mean ± SD (P values: *** <0.0001).

**Fig 7:**
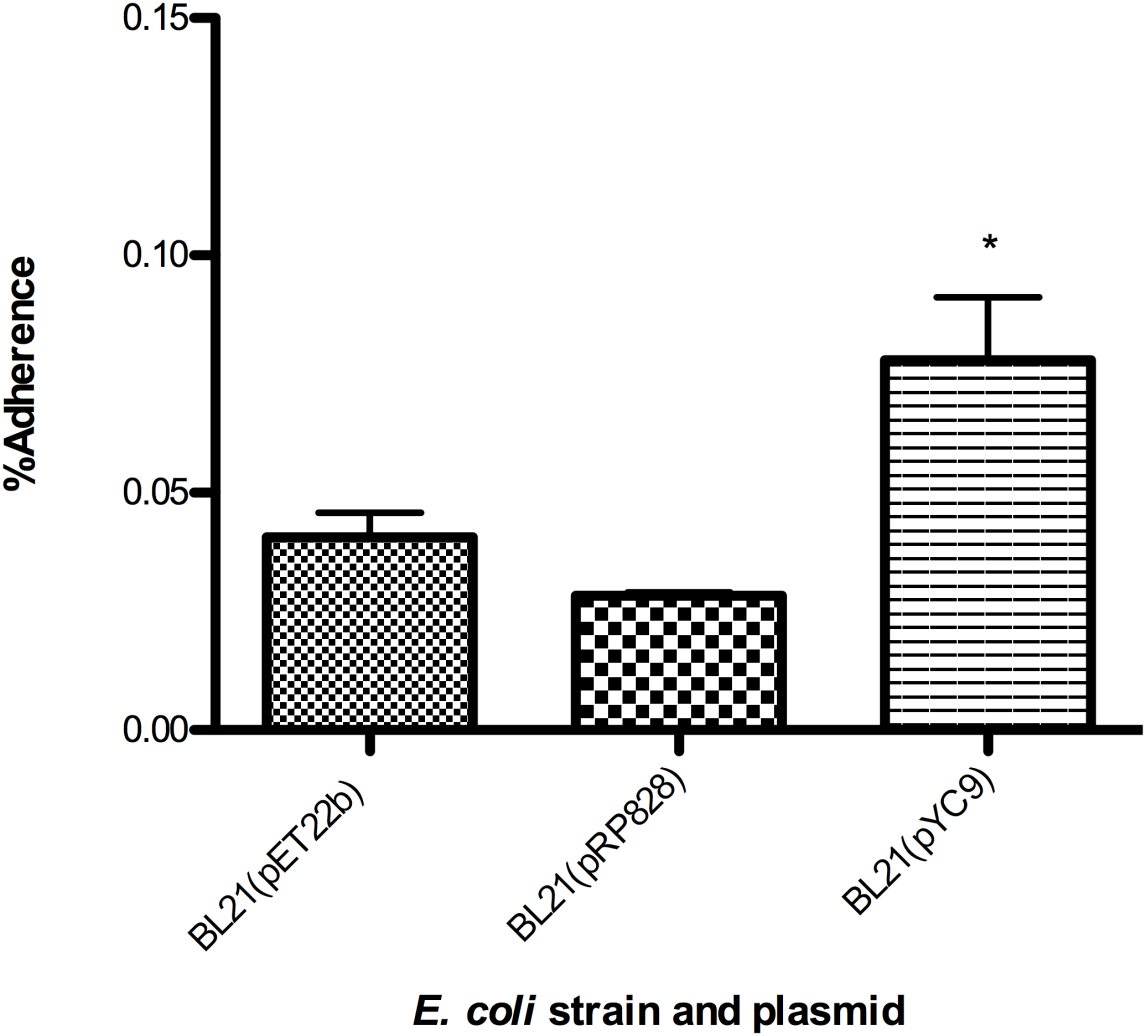
Adherence potential of *R*. *prowazekii* Adr2 expressing *E*. *coli*. Expression of *R*. *prowazekii* Adr2 in *E*. *coli* (BL21) is not sufficient to mediate cell adherence to EA.hy926 cells. The results are expressed as percentages of recovered adherent bacteria based on the total bacterial input. Results are shown as the mean ± SD (P values: * = 0.0123).

Previous studies have demonstrated that incubation of pathogenic Typhus Group (TG) rickettsial species with antiserum produced against Adr2 results in an overall reduction in infection of endothelial host cells [12, 15]. These findings suggested that *R*. *prowazekii* Adr2 may function as a potential adhesin and interacts with still unidentified human cell surface receptors. To test whether the *R*. *conorii* Adr2 homologue could similarly function as an adhesin, we employed a well-established heterologous expression system to examine the ability of Adr2-expressing *E*. *coli* to adhere to mammalian cells in the absence of serum proteins [6–11, 19, 20]. *E*. *coli* cultures expressing Adr2 were applied to confluent monolayers of human endothelial-like cell culture model (EA.hy926) cells and centrifuged to induce contact. Bacteria expressing the *R*. *conorii* OmpB protein (pYC9) or containing an empty vector (pET22b) were used as positive and negative controls, respectively. Following a brief incubation period, the cells were thoroughly washed free of non-adherent bacteria, and potential Adr2-mediated adherence determined by enumeration using a CFU-based quantification assay. As shown in Fig 6, there is no significant difference in the ability Adr2-expressing bacteria to adhere to endothelial cells when compared to *E*. *coli* harboring the empty vector, pET22b. In contrast, *E*. *coli* expressing the *R*. *conorii* protein, Sca5/OmpB [6] are adherent to these cells. In addition, we investigated whether the *R*. *prowazekii* Adr2 protein when expressed in a heterologous *E*. *coli* host was sufficient to mediate adherence to mammalian endothelial cells. As shown in Fig 7, similar to what was observed for the *R*. *conorii* homologue, the *R*. *prowazekii* Adr2 is not sufficient to confer adherence to mammalian cells when expressed at the outer-membrane of *E*. *coli*. Together, these data demonstrate that while independent expression of the *R*. *conorii* Adr2 and the *R*. *prowazekii* Adr2 proteins at the surface of *E*. *coli* is sufficient to contribute to evasion of complement-mediated killing, neither Adr2 protein is sufficient to mediate adherence to mammalian cells.

## Materials and methods

### Amino acid alignment and protein modelling

Amino acid sequences for Adr2 from *R*. *conorii* Malish 7 (gene identification number, gi: 499284784), *R*. *rickettsii* Sheila Smith (gi:157801598), *R*. *prowazekii* Madrid E (gi: 3861354) were aligned using ClustalW (Mac Vector 14.1). The *R*. *conorii* Adr2 sequence was analyzed by the Phyre2 protein structural prediction software. Secondary structures were predicted using Phyre2 modeling software.

### Antibodies

Anti-Adr2 polyclonal antiserum was generated from immunization of rabbits with peptides CNEEKDKATGVKLKSNTT, CKKSGAATNAPFTRTNISASHK, CTGLSSNTKNRTNISY, and CELAYSWIDDGRTKSKN corresponding to Adr2 amino acids 44–60, 90–110, 153–167, and 184–199 (loops 1, 2, 3, and 4 respectively) (Yenzym Antibodies). Total antibodies were recovered from antiserum utilizing HiTrapProteinA columns (GE Healthcare). Concentrations of the resulting antibodies were determined using Bradford assay. Polyclonal antibodies against *R*. *conorii* RplF have been described elsewhere [9].

### Plasmid assembly

Amplification of *adr2* from *R*. *conorii* Malish 7 genomic DNA flanked by a BamHI restriction site was accomplished using the primers 5’-GGATCCTGCAACAATGTTGTCTTCTA-3’ and 5’-GGATCCATATATCAAATCTTATACCG-3’. The resulting *adr2* DNA fragment was cloned into pCR2.1, digested with BamHI (New England Biolabs, Ipswich, MA), and ligated into the vector pET22b resulting in the construction of pSRK-2. pRP828 was constructed similarly using the primers 5’-GGATCCAATTTTATCTTCTAGTGTATCG-3’ and 5’-GGATCCGATATACCAAATCTTACACCTACTGT-3’. Construction of pJPO1, pAF17, pYC9 is described elsewhere [6, 9, 18]. A list of plasmids used in this study can be found in S1 Table.

### Culture of bacterial and mammalian cells

EA.hy926 and Vero (ATCC) cells were cultured in Dulbecco’s Modified Eagle Medium (Life Technologies) supplemented with 10% heat-inactivated FBS, 1% sodium pyruvate (Mediatech), and 1% MEM Non-Essential Amino Acids (Mediatech) at 37°C in 5% CO_2_ until used for experimentation.

*R*. *conorii* Malish 7 was propagated in Vero cells, needle-lysed and sucrose-gradient purified as previously described [21]. The resulting titers were enumerated as previously described [7]. *E*. *coli* BL21(DE3)(pET22b, pJP01, pYC9, pSRK-2) were grown overnight at 37°C in LB supplemented with carbenicillin. The bacteria were diluted 1:10 in fresh media, grown to OD_600_ ~0.5, and induced with 0.5mM IPTG for 4 h at 37°C. BL21(DE3)(pJP01) received 0.6mM IPTG.

### Bacterial fractionation

Outer membrane (OM) fractionations of *R*. *conorii* Malish 7 or *E*. *coli* BL21 (DE3) harboring either pET22b or pSRK-2 were prepared using sarkosyl solubilization as previously described [9, 22]. The isolated OM fractions were separated on SDS-PAGE and immunoblotted with the polyclonal rabbit anti-Adr2 described above and secondary antibody IRDye donkey anti-rabbit 800CW. In order to verify the efficiency of the fractionation, the lysates were probed for *E*. *coli* cytoplasmic contents using antibodies against *E*. *coli* RNA polymerase α-subunit. (4RA2, Neoclone). Additionally, *R*. *conorii* fractionations were probed with RplF antibody to verify the presence of cytosolic elements as previously described [9].

### Binding of host complement regulatory proteins

Induced *E*. *coli* BL21(DE3) cultures harboring pET22B, pSRK-2, or pJP01 were prepared as described above, and serum binding assays were performed as previously described [9]. Briefly, the bacterial cultures were incubated in either PBS or 50% NHS/PBS over ice in order to prevent complement-mediated killing. The samples were washed thoroughly with ice-cold PBS, lysed in 2X SDS-PAGE loading buffer, and separated by SDS-PAGE and then transferred to nitrocellulose membranes. Separated proteins on the nitrocellulose membranes were probed for vitronectin binding by western immunoblot analysis using rabbit anti-vitronectin (Complement Tech), and secondary antibody goat anti-rabbit-HRP(Sigma). Expression *R*. *conorii* Adr2 was confirmed using anti-His antibody (Genescript). Binding of multimeric vitronectin was assessed as previously described [18]. Cultures expressing Adr2 or Adr1 derivative protein were incubated with 25 ng/mL of multimeric vitronectin (Innovative Research) at room temperature for 1 hr. The samples were washed with PBS, separated by SDS-PAGE and analyzed by western immunoblot using the aforementioned anti-vitronectin (Complement Tech) and secondary goat anti-rabbit-HRP antibodies(Sigma). Expression of proteins was verified using mouse anti-His (Genescript).

### Cell adherence assay

Cell adherence assays were performed as previously described [6, 7]. Briefly, induced cultures of *E*. *coli* BL21 (DE3) harboring pET22B, pSRK-2, pRP828, or pYC9 were added to a confluent monolayer of EA.hy926 cells in serum-free media. To initiate adherence, a low speed centrifugation of 200xg for 5 min was performed and followed with a 20 min incubation at 37°C in the presence of 5% CO_2_. Cells were washed three times in PBS to remove non-adherent bacteria, and subsequently lysed with 0.1% Triton X-100 in sterile H_2_O. The resulting lysates were serially diluted in PBS and plated on LB agar to enumerate adhered bacteria. Results were expressed as a percentage of recovered bacteria based on the input inoculum.

### Serum resistance

*E*. *coli* BL21(DE3) cultures harboring pET22B, pSRK-2, or pJP01 were grown as described above. Cultures were washed with PBS and approximately 10^6^ colony forming units (cfu) were resuspended in either 200μl of PBS or 50% NHS/PBS and incubated for 1 h at 37°C. Following the incubation, the cultures were serially diluted in PBS and plated on LB-agar plates. The results were expressed as bacteria recovered (cfu) in PBS and 50%NHS/PBS following the incubation period and plotted on a logarithmic scale. These data represent independent triplicate samples, and the experiment was performed a minimum of three times.

### Flow cytometry analysis of Adr2

*R*. *conorii* or *E*. *coli* (BL21(DE3)) harboring pSRK-2 fixed with 4% PFA were used to probe for the presence of Adr2. Surface expression was detected using anti-Adr2 L1/L2 and secondary goat anti-rabbit-AlexaFluor488 (Molecular Probes). Bacteria were analyzed with a BD cytometer using FITC (488 nm excitation, 530/30 nm emission) and FloJo software.

### Statistical analysis of results

Data obtained for determination of serum resistance and cell adherence were analyzed by one-way ANOVA where significance was determined for p < 0.05.

## Discussion

In the present study, we describe an additional factor, Adr2, in two pathogenic rickettsial species that is sufficient to mediate resistance to serum-mediated killing [9, 10, 23]. We have demonstrated that recombinant Adr2 proteins from both *R*. *conorii* (RC1282) and *R*. *prowazekii* (RP828) are localized to the outer membrane when expressed in *E*. *coli* and are sufficient to confer resistance to innate deleterious effects of serum. We have additionally demonstrated the acquisition of fluid-phase host complement-regulatory vitronectin (Vn) by these expressed outer-membrane proteins.

Initial proteomic-based analysis of *R*. *prowazekii*(RP828) and *R*. *conorii* Adr2(RC1282) described a protein modeled after the *Neisseria meningitidis* NspA [24]. This initial analysis depicted a protein containing 10 trans-membrane β-strands and five extracellular domains. In contrast, our analysis using the Phyre2 online protein structural prediction server revealed a putative structure containing 8 trans-membrane regions and four extracellular domains (loops). However, it should noted that both of the predicted models reported were dependent on different algorithmic modeling software and are not based upon derived crystal structures [25]. Despite advances in crystallography studies to date, there are only a few solved crystal structures of proteins from rickettsial species [26–28]. As such, neither of these predicted models can be discarded until crystal structures of Adr2 proteins are solved.

Interestingly, while NspA was originally speculated to function similarly to the related opacity proteins (Opas) as an adhesin, recent data has revealed that NspA also functions as a serum-resistance factor by binding complement regulatory protein, Factor H (fH) [29]. We have also demonstrated the deposition of another regulatory protein, vitronectin (Vn), to surface-expressed *R*. *conorii* Adr1 and that this interaction is crucial to survival of this pathogen in the presence of normal human serum. Vitronectin is a multi-faceted regulatory protein that exists in human extracellular matrix and serum. Within the serum, Vn plays a role in cell adhesion, hemostasis, and regulating the formation of the terminal complement complex which results in the lysis of target cells [30]. We have demonstrated that both SFG and TG rickettsial species likely possess redundancy in the mechanisms by which this class of pathogens subverts complement-mediated killing [7]. Elucidating the various strategies evolved by pathogenic bacteria in order to evade this innate immune clearance from the blood circulation may reveal conserved proteins that could function as candidates for therapeutic intervention, which has recently been described [13, 14].

As seen in the Adr2 alignment in Fig 1A, the predicted extracellular motifs are well conserved among SFG and TG rickettsial species. Interestingly, the predicted Adr2 extracellular moieties 3 and 4 contain an overabundance of positively charged residues (23% and 18% respectively), as has been previously reported for the predicted extracellular loops 3 and 4 of Adr1 [7]. The analogous regions in *R*. *conorii* Adr1 were sufficient to not only confer serum resistance, but also demonstrated the ability to sequester Vn when expressed in *E*. *coli*. Indeed, a recent study examining Vn binding by various human pathogenic bacteria revealed a potential role of electrostatic interactions in this crucial protein-protein interaction [31]. Whether the lysine and other positively charged residues within extracellular Adr2 domains also play a role in Adr2-Vn binding and subsequent serum resistance is an area of current investigation.

Serum analysis of patients with active *R*. *conorii* infections reveals an increase in markers of complement activation [32]. We posit that, even in the face of an activated complement cascade, *Rickettsia* species are resistant to complement-mediated killing [9–11, 18]. Accordingly, the studies described herein contribute to our understanding of rickettsial resistance to complement mediated killing. Future analysis will be needed to elucidate the relationship between complement activation and rickettsial pathogenesis.

It has previously been reported that both *R*. *prowazekii* Adr2 and *R*. *conorii* Adr2 interact with unidentified surface-exposed mammalian cell proteins, suggesting that Adr2 may function as an adhesion [25]. In this work, we examined the potential of both Adr2 proteins to mediate adhesion to host cells using our gain-of-function *E*. *coli* heterologous expression system. When either Adr2 from *R*. *conorii* or *R*. *prowazekii* is expressed at the surface of *E*. *coli*, this protein does not promote adherence to host cells as compared to *E*. *coli* harboring an empty vector. It is possible that in *E*. *coli*, recombinant Adr2 proteins do not fold into the same structure at the outer-membrane as on the surface of native rickettsial species. However, we would argue that while possible, this scenario is not likely as recombinant Adr2 proteins assemble at the outer-membrane and retain the ability to mediate interactions with Vn and contribute to serum resistance. Recombinant Adr2 does not mediate adherence using this experimental system; however, this phenotype does not preclude additional functions of Adr2 at the surface of both intact *R*. *conorii* and *R*. *prowazekii* cells. Whether the putative adhesin function or the Vn-binding function of Adr2 proteins contribute to pathogenesis remains to be elucidated.

## Supporting information

S1 Table List of plasmids. (TIF)
