# Supplementary figures and images for: Expression of Rickettsia Adr2 protein in E. coli is sufficient to promote resistance to complement-mediated killing, but not adherence to mammalian cells
Source: PLoS One. 2017 Jun 29;12(6):e0179544. doi: 10.1371/journal.pone.0179544 (PMC5491016; doi:10.1371/journal.pone.0179544)

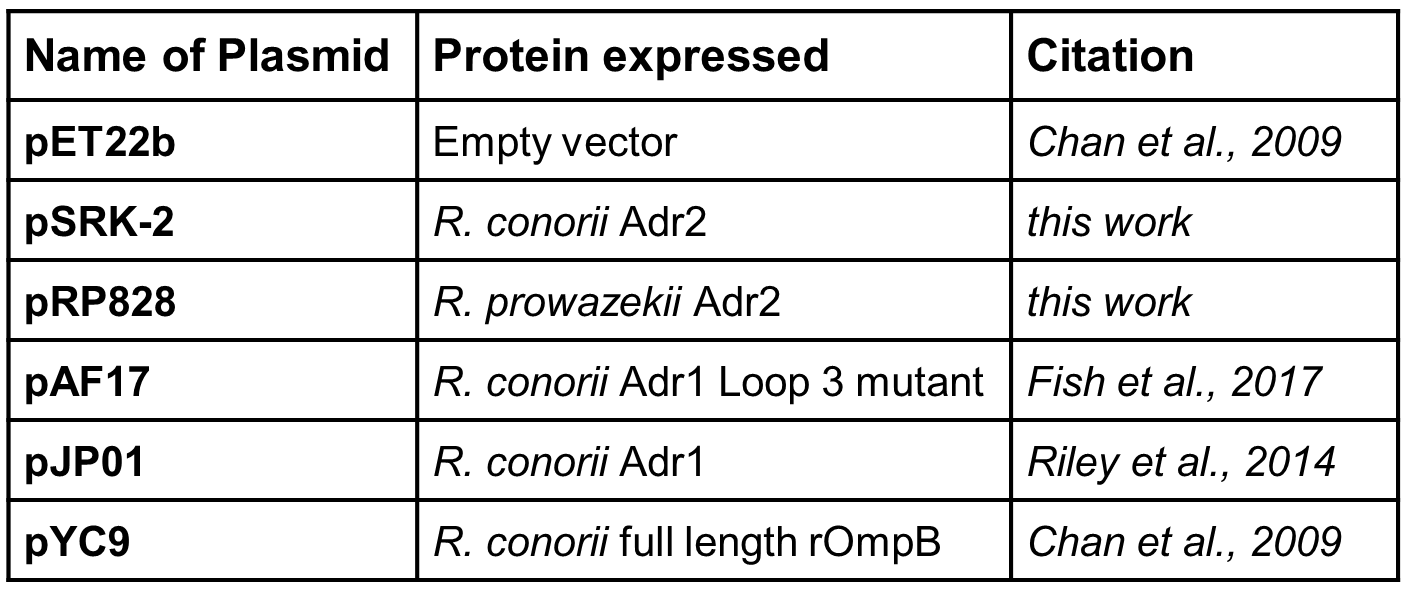

Supplement: S1 Table — (TIF) [file pone.0179544.s001.tif]
